# Regulation of *sod1* mRNA and protein abundance by zinc in fission yeast is dependent on the CCR4-NOT complex

**DOI:** 10.1016/j.jbc.2025.108156

**Published:** 2025-01-04

**Authors:** Andrew T. Weeks, Amanda J. Bird

**Affiliations:** 1 Department of Human Nutrition, Ohio State University, Columbus, Ohio, USA; 2 Department of Molecular Genetics, Ohio State University, Columbus, Ohio, USA; 3 Center for RNA Biology, Ohio State University, Columbus, Ohio, USA

**Keywords:** zinc, zinc homeostasis, nutrient deficiency, superoxide dismutase, post-transcriptional regulation, amyotrophic lateral sclerosis (ALS)

## Abstract

Zinc is an essential micronutrient that serves as a cofactor in a wide variety of enzymes, including Cu-Zn Superoxide Dismutase 1 (Sod1). We have discovered in *Schizosaccharomyces pombe* that Sod1 mRNA and protein levels are regulated in response to cellular zinc availability. We demonstrate that lower levels of *sod1* mRNA and protein accumulate under low zinc conditions and that this regulation does not require the *sod1* promoter or known factors that regulate the transcription of *sod1* in response to zinc and other environmental stresses. Further analyses using yeast deletion strains and an inactive allele of Caf1 revealed that the reduced accumulation of *sod1* mRNA and protein under low zinc conditions depends on the Caf1 and Ccr4 deadenylases of the CCR4-NOT complex. We also found that Caf1 and Ccr4 are both required for growth under zinc-limiting conditions. To gain additional mechanistic insight we used immunoblot analysis to map the regions required for the regulation of the Sod1 protein by zinc. We found that the *sod1* ORF and 3′UTR are both necessary and sufficient for the zinc-dependent changes in Sod1 protein abundance. Our studies reveal a novel mechanism of altering mRNA and protein abundance in response to zinc status, which depends on the CCR4-NOT complex.

Zinc is an essential nutrient that serves as a catalytic cofactor for over 300 enzymes that are involved in a wide range of cellular processes, including translation, protein biosynthesis and folding, glycolysis, amino acid metabolism, and vesicle-mediated transport (1, 2). Zinc is also critical for the formation of structural domains such as C_2_H_2_-type zinc fingers, which commonly facilitate interactions with DNA, RNA, and proteins (3, 4). In addition to being important for the survival of all living organisms, excess zinc can be toxic to cells (5). Because of these essential and potentially toxic properties, all cells rely on mechanisms to tightly regulate intracellular zinc ion concentrations. The importance of these mechanisms is highlighted by the fact that multiple diseases result from genetic mutations that disrupt zinc transporter function (6, 7, 8). Imbalances in zinc levels have also been documented in complex diseases such as Alzheimer’s disease, certain cancers, and type 2 diabetes (9, 10, 11).

In both prokaryotes and eukaryotes, zinc-responsive transcription factors play a primary role in zinc homeostasis by regulating the expression of zinc transporter genes and other genes related to zinc ion availability or its distribution (12, 13, 14). While many studies have focused on transcriptional responses to zinc, multiple zinc transporters have been identified that are regulated at post-transcriptional levels in response to zinc availability. In *Arabidopsis*, intron retention in a ZIF2 splice variant enhances translation under excess zinc conditions, leading to increased levels of the ZIF2 vacuolar zinc transporter and increased tolerance to zinc (15). Excess zinc enhances the stability of the human ZNT5 mRNA (16), whereas the ZIP4 mRNA is stabilized by zinc deficiency, which ensures optimal zinc absorption when the body requires zinc (17, 18). While post-transcriptional control mechanisms clearly contribute to cellular zinc homeostasis, it is unclear how mRNA stability is influenced by cellular zinc status and the extent to which these mechanisms are conserved in other organisms.

In this study we used the fission yeast *Schizosaccharomyces pombe* as a model system to identify new mechanisms by which cells adjust gene expression to optimize growth in low zinc conditions. In *S. pombe*, the Loz1 transcriptional repressor plays a central role in maintaining zinc homeostasis by regulating the expression of ∼30 genes in response to zinc availability (19, 20, 21). As Loz1 functions as a repressor in high zinc, Loz1 target genes are typically expressed at high levels when zinc is limiting and are expressed under both low and high zinc conditions in a *loz1* deletion strain. In RNA-seq analysis to identify *Loz1* target genes, we found that the expression of eight mRNAs was reduced under zinc-deficient conditions, three of which were regulated independently of *Loz1* (20). Among these three, two (*sod1* and *SPCP20C8.03*) were also reported in other studies as mRNAs with reduced expression under low-zinc conditions (22, 23). To uncover novel mechanisms by which gene expression is regulated by zinc, here we investigated how *sod1* mRNA levels are regulated by zinc in *S. pombe*. We focused on *sod1* because its expression is zinc-regulated in other organisms, although the mechanisms remain unknown (24). We show that *sod1* mRNA and protein levels are reduced in zinc-deficient cells and that the Caf1 and Ccr4 deadenylases from the conserved CCR4-NOT complex are required for this regulation. We also find that the regulation of Sod1 protein abundance depends on both the *sod1* ORF and 3′UTR region, indicating the presence of a novel mechanism to optimize gene expression in response to cellular zinc status.

## Results

### Sod1 mRNA levels are regulated by zinc in a manner that is independent of Loz1 and other stress response pathways

**Figure 1:**
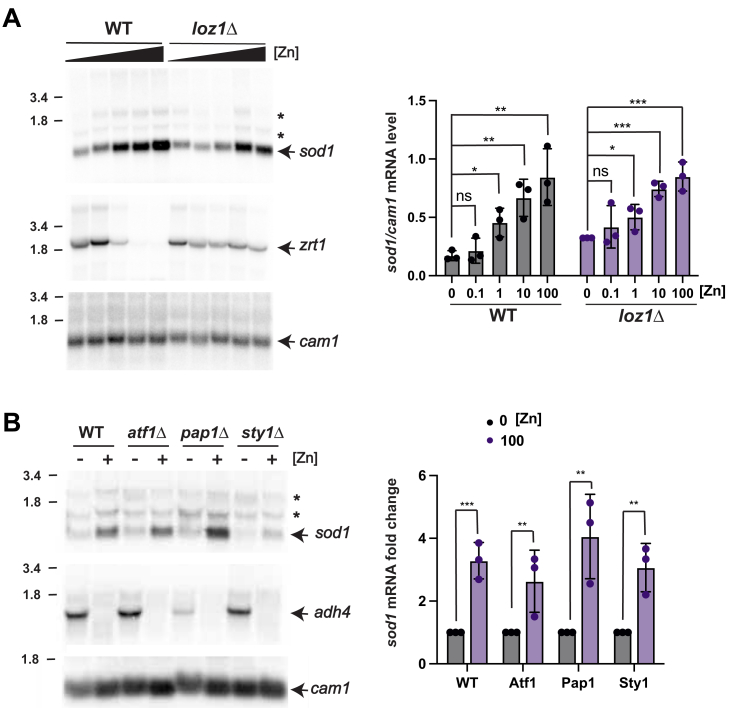
***sod1* mRNA abundance is regulated by zinc.***A*, total RNA was extracted from wild-type (WT) and *loz1*Δ cells grown in ZL-EMM supplemented with 0, 0.1, 1, 10, or 100 μM zinc. A representative RNA blot is shown probed for *sod1*, zinc-limiting control *zrt1*, and loading control calmodulin (*cam1*). The major sod1 transcript of ∼0.8 kb is indicated by an *arrow*. Quantification of 3 independent RNA blots is shown on the *right*. The values are the means of 3 replicates ± standard deviations. *B*, total RNA was extracted from the indicated strains grown in ZL-EMM without (−Zn) or with (+Zn) 100 μM zinc. Shown is a representative RNA blot probed for *sod1*, zinc-limiting control *adh4*, and loading control *cam1*. The quantification of 3 independent blots is shown on the *right*, where *sod1*/*cam1* levels were normalized to 0 zinc. Mean values are shown with error bars ± standard deviations. *p* values were determined using two-tailed unpaired Student's *t* test. ∗*p* < 0.05, ∗∗*p* < 0.01, ∗∗∗*p* < 0.001.

Previous RNA seq. analysis in *S. pombe* suggested that *sod1* mRNA abundance is altered in response to zinc levels and that this regulation was independent of the zinc-responsive transcriptional repressor Loz1 (20). To directly test whether *sod1* gene expression is regulated by zinc independently of Loz1, we used RNA blot analysis to examine the abundance of *sod1* transcripts in wild-type (WT) and *loz1*Δ cells grown overnight in ZL-EMM supplemented with a range (0–100 μM) of Zn^2+^ (Fig. 1*A*). As a control, we measured the expression of Zrt1, a high-affinity zinc transporter whose expression is Loz1 dependent. Using a strand-specific *sod1* probe, we detected a strong zinc-regulated transcript of approximately 0.8 kb, consistent with the predicted size of the *sod1* mRNA (763 nucleotides) (25) (Fig. 1*A* arrow). We also detected lower levels of multiple larger transcripts that were not regulated by zinc (Fig. 1*A* asterisks). The significance of these transcripts is discussed later in the text. The quantification of the 0.8kb *sod1* transcript revealed a 4-fold reduction in *sod1* mRNA abundance in wild-type cells and a 2.6-fold reduction in *loz1*Δ cells under low zinc compared to high zinc conditions. By contrast, *zrt1* mRNA abundance was repressed in response to zinc in wild-type cells but accumulated in low and high zinc conditions in the *loz1* deletion mutant. These results show that the regulation of *sod1* by zinc differs from that of *zrt1*, suggesting the presence of a novel regulatory mechanism.

In *S. pombe*, *sod1* is regulated by the conserved core environmental stress response (CESR), which is dependent on the Win1-Wis1-Sty1 SAPK pathway and the Atf1 and Pap1 transcription factors (26, 27). To determine if components of the CESR pathway are necessary for the zinc-dependent changes in *sod1* expression, yeast strains with deletions of *atf1*, *pap1*, or *sty1* were grown overnight in ZL-EMM supplemented with 0 or 100 μM Zn^2+^, and *sod1* mRNA abundance examined by RNA blotting (Fig. 1*B*). RNA blots were also probed for the Loz1-regulated *adh4* mRNA (19). Sod1 mRNA levels decreased by 2.6- to 4.1-fold in low zinc in the three CESR deletion strains, indicating that the regulation is independent of the core environmental stress response pathway.

**Figure 2:**
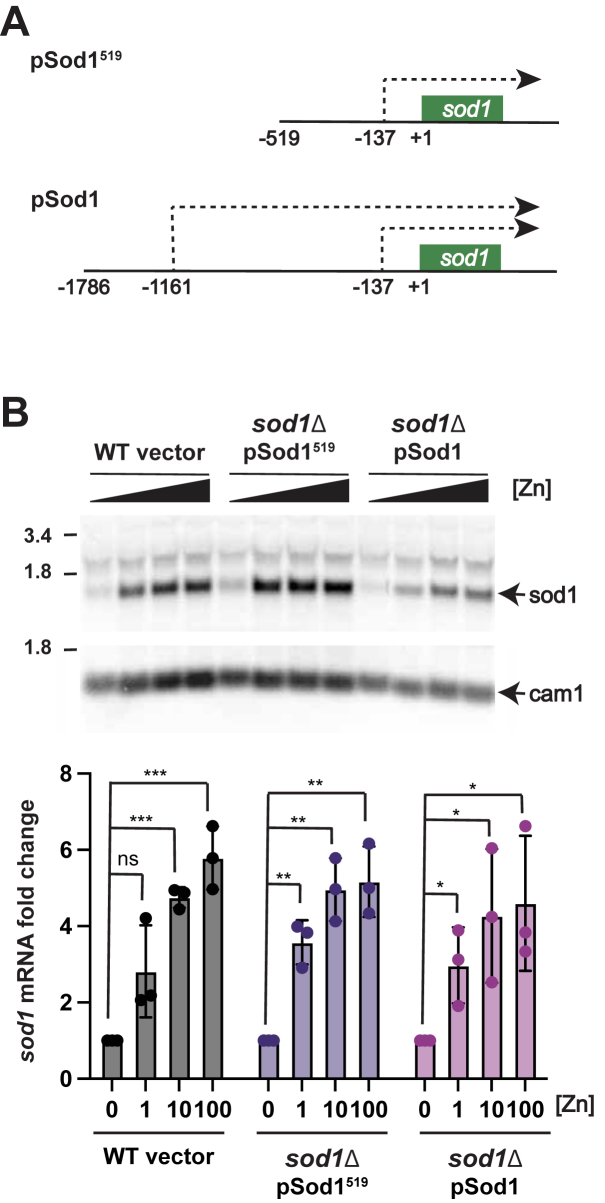
**Plasmids containing the *sod1* gene and promoter are sufficient for zinc-dependent regulation of *sod1* mRNA abundance.***A*, schematic diagram of the pSod1^519^ and pSod1 plasmids. The positions of previously mapped TSSs are indicated (*dotted lines*), with positions relative to the first base of the translational initiation codon (numbered as +1). *B*, total RNA was extracted from wild-type cells with the empty vector, and *sod1*Δ cells expressing pSod1^519^ and pSod1, following growth overnight in ZL-EMM with 0, 1, 10, or 100 μM Zn^2+^. A representative RNA blot is shown, probed for *sod1* and loading control *cam1*. The quantification of the *sod1/cam1* ratio normalized to 0 zinc is shown below. Values are the means of three independent biological replicates ± standard deviations. *p* values were determined using a two-tailed unpaired Student's *t* test. ∗*p* < 0.05, ∗∗*p* < 0.01, ∗∗∗*p* < 0.001.

As the regulation of the *sod1* mRNA was independent of Loz1, and other factors known to regulate *sod1* expression, we set out to determine which region(s) of the *sod1* gene and promoter were necessary and sufficient for the zinc-dependent regulation. Cap Analysis of Gene Expression (CAGE) in fission yeast has shown that under standard growth conditions the *sod1* mRNA is initiated at a transcriptional start site 137 nucleotides (nt) upstream of the translational AUG start (28). However, as cells enter the stationary phase, there is a shift to the production of a longer *sod1* transcript that is initiated 1161 nucleotides upstream of the AUG (28, 29). Based on these published results and the identification of longer transcripts with the *sod1* probe in RNA blot analysis (Fig. 1, *A* and *B*, asterisks), we generated two plasmids containing the *sod1* ORF and 3′UTR, with either 1786 bp or 519 bp of upstream promoter sequences, which included or excluded the transcriptional start site for the longer *sod1* transcript, respectively (Fig. 2*A*). Each plasmid was integrated into the genome of a *sod1* deletion strain, and the regulation of *sod1* expression in response to zinc was examined by RNA blot analysis (Fig. 2*B*). We observed an ∼5-fold decrease in the 0.8 kb *sod1* transcript abundance in low zinc vs. high in *sod1*Δ cells expressing the pSod1^519^ or pSod1 plasmids. We therefore conclude that the DNA sequences contained within both plasmids are sufficient for the regulation of *sod1* expression by zinc. We also noted that there was no change in the size or abundance of the larger transcripts in wild-type, *sod1*Δ pSod1^519^, and *sod1*Δ pSod1 cells (see Fig. 2*B* asterisks). As the pSod1^519^ plasmid does not include the upstream regulatory region required to produce the longer *sod1* transcripts that were previously documented in stationary phase cells, we conclude that these higher molecular weight bands detected in our analyses are non-specific.

In our previous studies, we had found that the activity of a *sod1-lacZ* reporter which contained the longer 1786 bp *sod1* promoter region, including the *sod1* 5′UTR fused to the *lacZ* gene, was not regulated when cells were grown in ZL-EMM with or without 200 μM Zn^2+^ (19). Since our current growth conditions examined mRNA levels in 0 and 100 μM zinc, we examined the activity of the *sod1-lacZ* reporter under these conditions (Fig. S1). There was no difference in β-galactosidase activity in low vs. high zinc conditions in cells expressing the *sod1-lacZ*, consistent with our previous observations that the *sod1* promoter is not sufficient for the zinc-dependent regulation of *sod1* transcript abundance.

### The regulation of *sod1* mRNA abundance by zinc requires Caf1 and Ccr4

**Figure 3:**
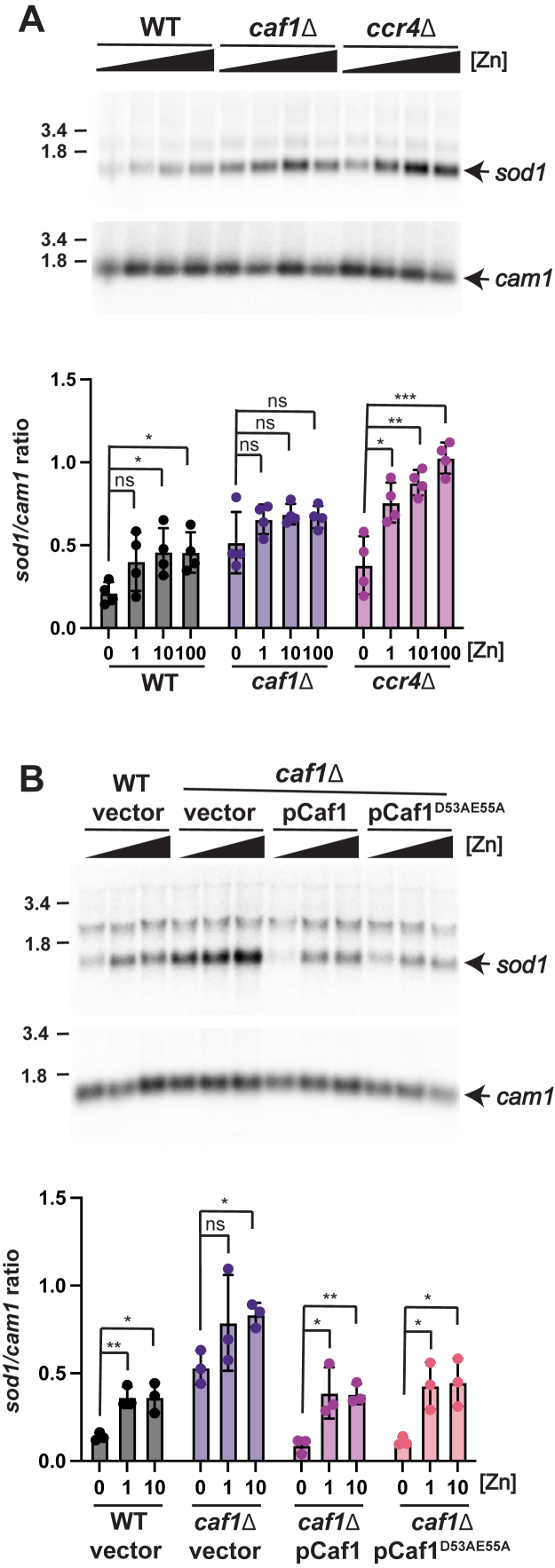
**Caf1 and Ccr4 have redundant roles in the zinc-dependent regulation of *sod1* mRNA abundance.***A*, total RNA was extracted from wild-type (WT), *caf1*Δ, and *ccr4*Δ cells grown overnight in ZL-EMM with either 0, 1, 10, or 100 μM zinc for RNA blot analysis. A representative RNA blot is shown probed for *sod1* and loading control *cam1*. Quantification is shown below where *sod1* levels were normalized to *cam1.* The results show the mean of four independent biological replicates ± standard deviations. *B*, total RNA was extracted from wild-type (WT) with an empty vector, and *caf1*Δ, cells expressing the indicated plasmid following growth overnight in ZL-EMM with 0, 1, or 10 μM zinc for RNA blot analysis. Blots were probed and quantified as described in panel A. *p* values were determined using a two-tailed unpaired Student's *t* test. ∗*p* < 0.05, ∗∗*p* < 0.01, ∗∗∗*p* < 0.001.

Since the regulation of *sod1* mRNA was independent of the promoter, we hypothesized that *sod1* mRNA levels were regulated at a post-transcriptional level in response to zinc. In yeast, the degradation of most mRNAs is initiated by deadenylation, which requires the CCR4-NOT complex (30). Poly (A) tail shortening then triggers 3′-5′ decay by the cytoplasmic exosome, or decapping and subsequent 5′-3′ decay (31, 32). In fission yeast, the CCR4-NOT complex consists of eight main subunits, including the Ccr4 and Caf1 deadenylases (33). To test whether *sod1* mRNAs are preferentially degraded in low-zinc conditions in a manner that is dependent on the CCR4-NOT complex, wild-type and deletion strains of *caf1* and *ccr4* were grown overnight in ZL-EMM with or without Zn^2+^. *sod1* mRNA abundance was then analyzed by RNA blot analysis (Fig. 3*A*). Compared to the wild-type, deletion of *caf1* led to an increase in *sod1* mRNA abundance under all conditions and resulted in the loss of zinc-dependent differences in *sod1* mRNA abundance. Deletion of *ccr4* also led to an increase in total *sod1* mRNA abundance in all growth conditions. However, in the *ccr4* deletion strain, *sod1* mRNA abundance was still regulated by zinc status.

The above results revealed that deletion of *caf1* led to a loss of zinc-dependent regulation of *sod1*, whereas deletion of *ccr4* did not. Within the CCR4-NOT complex, Caf1 tethers Ccr4 to the complex, facilitating its integration and function (34). Since deletion of *caf1* results in the loss of both Ccr4 and Caf1 from the CCR4-NOT complex, we tested whether the phenotype of the *caf1* mutant was due to the loss of Caf1 deadenylase activity alone or the combined loss of both Caf1 and Ccr4 activities. For this, a catalytically inactive Caf1 plasmid was constructed by introducing substitutions (D53A and E55A) that disrupt Caf1 activity, without affecting its ability to tether Ccr4 to the CCR4-NOT complex (35, 36). When *sod1* transcript abundance was examined in *caf1*Δ cells expressing either the catalytically active (pCaf1) or inactive (pCaf1^D53AE55A^) Caf1 plasmid, both the total *sod1* mRNA level and the zinc-dependent differences in *sod1* abundance were similar to those in wild-type cells (Fig. 3*B*). As the inactive Caf1^D53AE55A^ allele restores *sod1* mRNA abundance in *caf1*Δ to levels similar to those in wild-type cells, these results indicate that the Ccr4 deadenylase is sufficient for the zinc-dependent regulation of the *sod1* mRNA.

### The regulation of Sod1 protein abundance by zinc requires Caf1 and Ccr4

**Figure 4:**
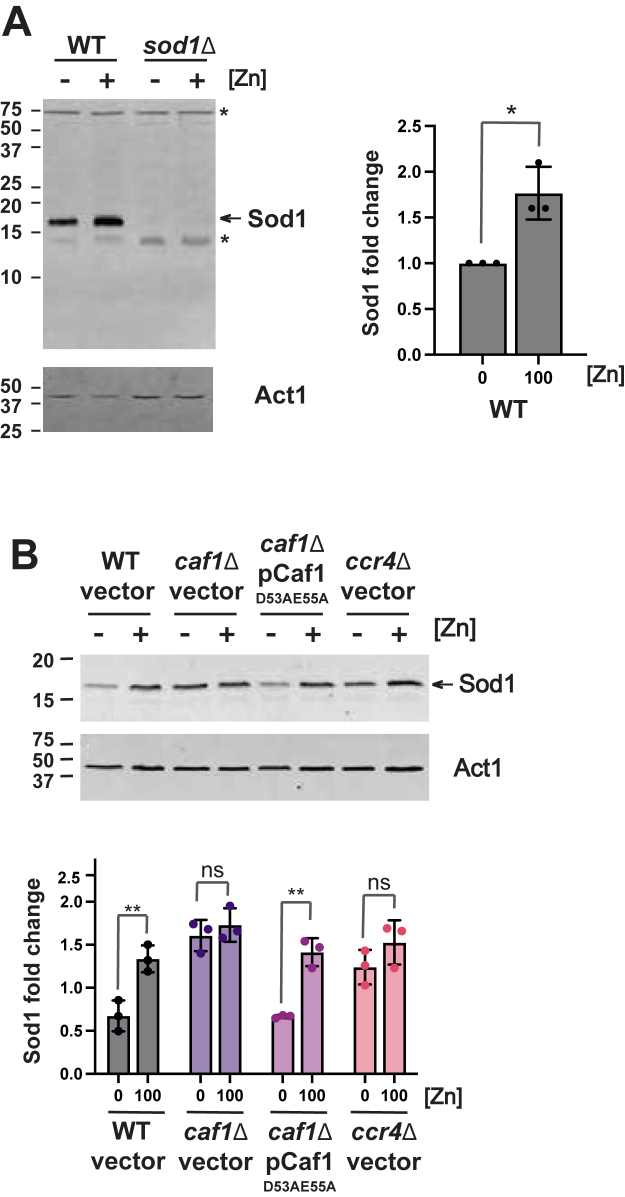
**The regulation of Sod1 protein abundance by zinc is dependent upon Caf1 and Ccr4.***A*, total protein was extracted from wild-type cells grown in zinc-limiting EMM (ZL-EMM) supplemented with 0 or 100 μM zinc and was analyzed by immunoblot analysis. Total protein extracts were also prepped from *sod1*Δ cells that were grown in ZL-EMM with 0 or 10 μM zinc, as this strain is unable to grow at higher zinc concentrations. Immunoblots were hybridized with antibodies to Sod1 and loading control Act1 (Actin). Non-specific bands are marked with an asterisk. Molecular weight markers in KDa are indicated on the *left*. The quantification is shown in the *right panel*, where Sod1/Actin levels were normalized to 0 zinc. Values are the means of 3 independent biological replicates with error bars showing ± standard deviations. *B*, immunoblots were performed as described above using total protein extracted from wild-type, *caf1*Δ, and *ccr4*Δ with the indicated plasmids. The quantification of Sod1 signal normalized to Actin is shown below. The results show the mean value of 3 independent biological replicates ± standard deviations. *p* values were determined using two-tailed unpaired Student's *t* test. ∗*p* < 0.05, ∗∗*p* < 0.01.

To determine if Sod1 protein levels were also regulated by zinc, we isolated total protein extracts from wild-type and *sod1*Δ cells grown overnight in ZL-EMM with 0 or 100 μM Zn^2+^ for immunoblot analysis. When immunoblots were probed with antibodies to Sod1, a strong band of ∼17kDa was detected in wild-type extracts, which was absent in extracts from the *sod1* deletion strain (Fig. 4*A*). We also detected two non-specific bands at ∼15kDa and ∼75kDa in wild-type and *sod1*Δ extracts (see Fig. 4*A* asterisks). The abundance of the Sod1-specific band decreased by 1.8-fold in zinc-deficient cells, indicating that Sod1 protein levels are dependent on zinc status.

We next tested whether Sod1 protein abundance was affected by the deletion of *ccr4* or *caf1*, or the inactivation of Caf1. For the most part, Sod1 protein levels mirrored *sod1* mRNA levels. Deletion of *caf1* led to high levels of Sod1 protein in both low and high zinc conditions, while Sod1 protein levels were regulated by zinc in *caf1*Δ cells expressing pCaf1^D53AE55A^ (Fig. 4*B*). An exception was observed in *ccr4*Δ cells, where *sod1* mRNA levels were regulated by zinc (Fig. 3*A*), but Sod1 protein levels were not (Fig. 4*B*). This suggests that Sod1 protein may be subject to additional regulation at a translational level.

### The *sod1* ORF and 3′UTR are sufficient for zinc-dependent changes in Sod1 protein abundance

**Figure 5:**
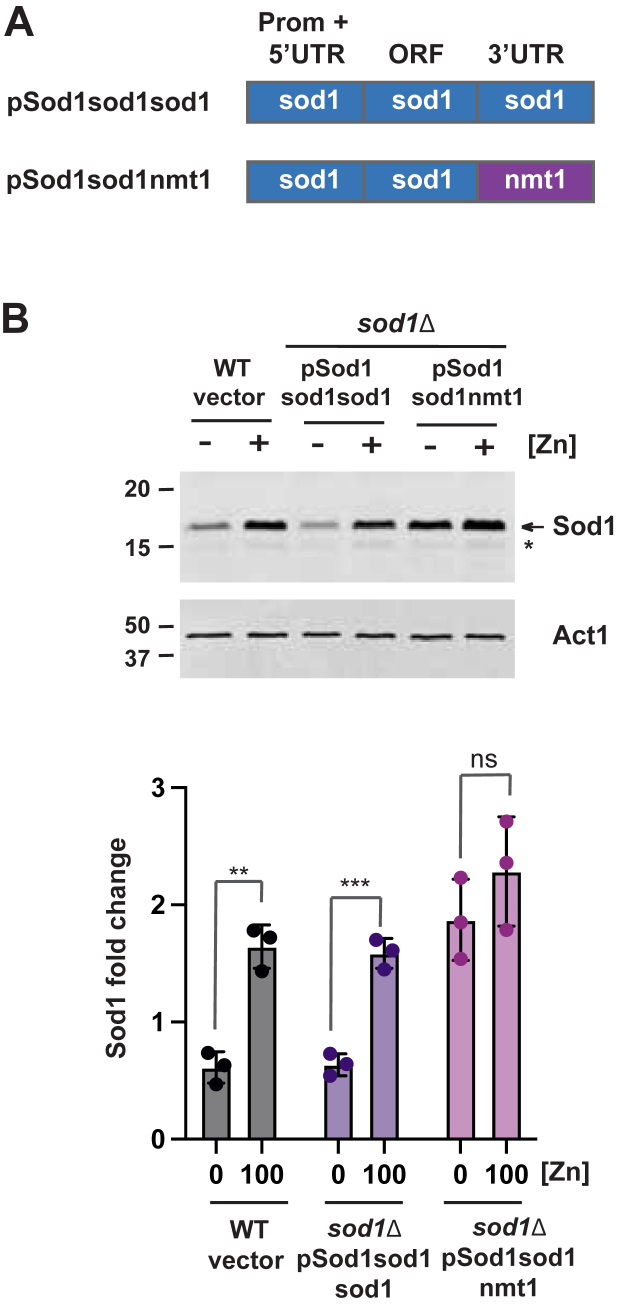
**The *sod1* 3′ UTR is required for zinc-dependent changes in Sod1 protein abundance.***A*, Schematic representation of *sod1* plasmids containing the *sod1* promoter and ORF with either the *sod1* or *nmt1* 3′ UTR. *B*, immunoblots were performed with total protein extracts from wild-type cells with the empty vector and *sod1*Δ strains expressing the indicated plasmids grown in zinc-limiting EMM (ZL-EMM) supplemented with 0 or 100 μM zinc. Immunoblots were hybridized with antibodies to Sod1 and loading control Actin (Act1). The quantification of the Sod1 signal normalized to Actin is shown below with the values representing the mean of 3 independent biological replicates ± standard deviations. *p* values were determined using two-tailed unpaired Student's *t* test. ∗∗*p* < 0.01, ∗∗∗*p* < 0.001.

To gain additional insight into the regulation of Sod1 by zinc, we used immunoblot analysis to map the regions of the *sod1* gene required for regulating Sod1 protein levels. To determine if the *sod1* 3′UTR is required for regulation, we generated *sod1* plasmid constructs that expressed the *sod1* ORF from the longer *sod1* ∼1.8 kb promoter region, either with the *sod1* 3′UTR or a control *nmt1* 3′UTR (Fig. 5*A*). When Sod1 protein abundance was compared in *sod1*Δ cells expressing these plasmids, Sod1 protein levels were regulated by zinc in cells containing plasmids with the *sod1* 3′UTR, but not in those with the *nmt1* 3′UTR (Fig. 5*B*). These results indicate that the *sod1* 3′UTR is necessary for the zinc-dependent regulation of Sod1 protein levels.

**Figure 6:**
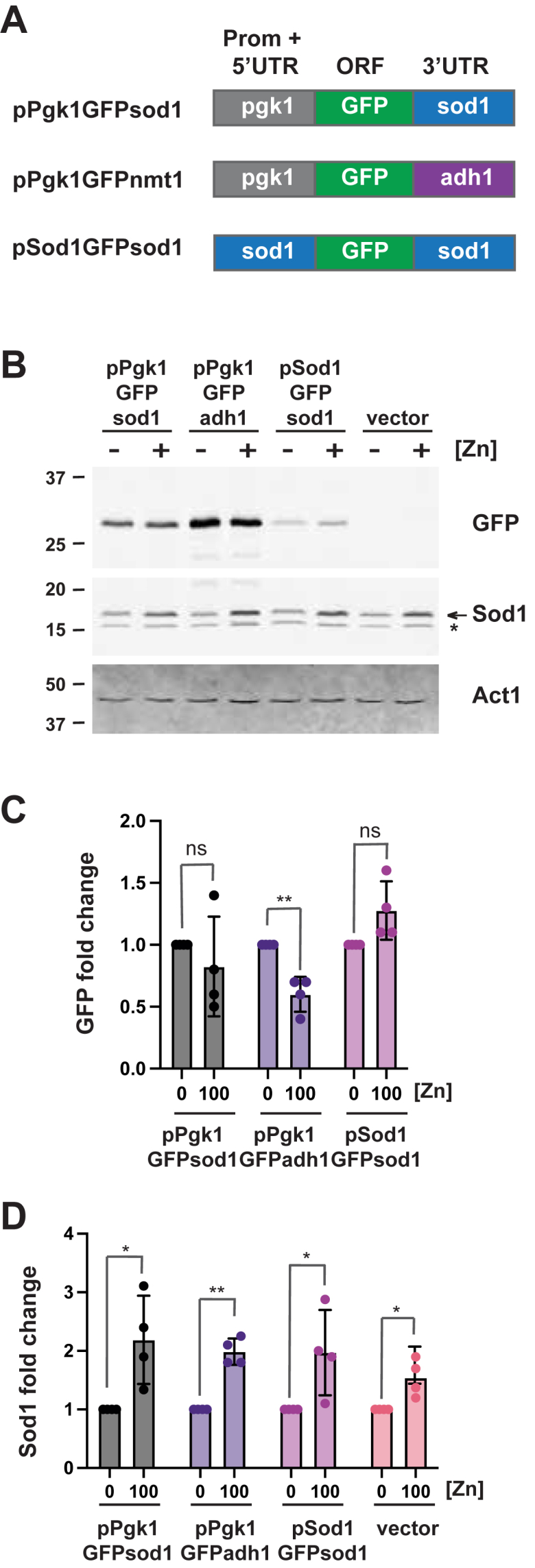
**The *sod1* 3′ UTR is insufficient for zinc-dependent changes in Sod1 protein abundance.***A*, schematic representation of GFP reporter constructs containing the *sod1* or *adh1* 3′ UTRs fused to GFP under control of the *pgk1* or *sod1* promoters. *B*, immunoblot analysis using total protein extracts from wild-type cells with the empty vector or expressing the indicated GFP plasmids grown in zinc-limiting EMM (ZL-EMM) supplemented with 0 or 100 μM zinc. Immunoblots were hybridized with antibodies to Sod1, GFP, and loading control Actin (Act1). Non-specific bands are marked by an *asterisk*. *C and D*, the quantification of 3 independent blots with the GFP or Sod1 signals normalized to Actin. Values represent the mean of the three independent biological replicates ± standard deviations. *p* values were determined using two-tailed unpaired Student's *t* test. ∗*p* < 0.05, ∗∗*p* < 0.01.

To test whether the *sod1* 3′UTR was sufficient for zinc-dependent changes in Sod1 protein abundance, we generated plasmid constructs that expressed GFP from the *pgk1* promoter, either with the *sod1* 3′UTR or a control *Saccharomyces cerevisiae adh1* 3′UTR (Fig. 6*A*). Both plasmids were integrated into the genome of wild-type cells. The levels of GFP and the endogenous Sod1 protein were then examined by immunoblot analysis following growth of cells in ZL-EMM with 0 or 100 μM Zn^2+^ (Fig. 6, *B* and *C*). The levels of the endogenous Sod1 protein were measured to confirm that cells were zinc-limited or zinc-replete. In cells expressing pPgk1GFPsod1, GFP accumulated to similar levels under both low- and high-zinc conditions, suggesting that the *sod1* 3′UTR alone is not sufficient to confer the zinc-dependent regulation of Sod1 protein abundance. Since overexpression of *sod1* from the *pgk1* promoter might interfere with the regulatory mechanism, we also generated an additional GFP construct where the *pgk1* promoter was replaced with the ∼1.8kb *sod1* promoter. This substitution did not result in the zinc-dependent regulation of GFP. Taken together, we conclude that while the *sod1* 3′UTR is required for the zinc-dependent regulation of Sod1 protein abundance, it is not sufficient on its own to confer this regulation.

**Figure 7:**
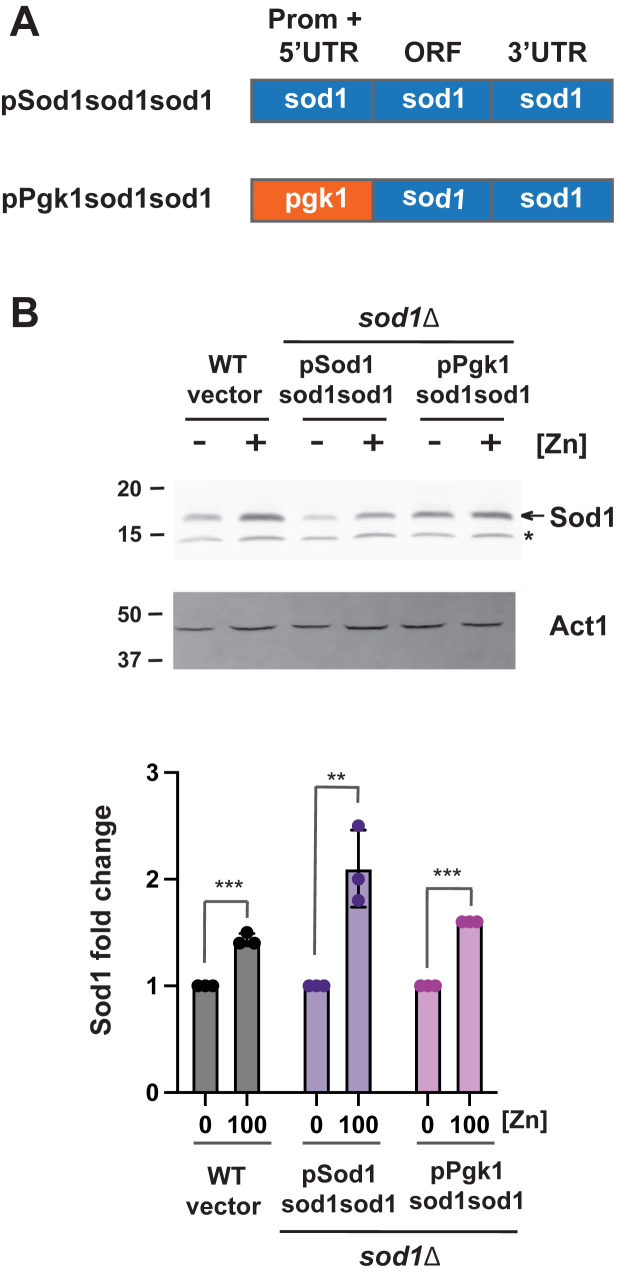
**The *sod1* ORF and 3′ UTR are sufficient for zinc-dependent changes in Sod1 abundance.***A*, schematic representation of constructs containing the *sod1* ORF and 3′ UTR under control of either the *sod1* or *pgk1* promoter. *B*, total protein was extracted from wild-type cells with empty vector and *sod1*Δ strains expressing the indicated plasmids following growth in zinc-limiting EMM (ZL-EMM) with 0 or 100 μM zinc for immunoblot analysis. Immunoblots were hybridized with antibodies to Sod1 and loading control Actin (Act1). Non-specific bands are marked by an *asterisk*. Quantification of 3 independent blots with Sod1 signal normalized to Actin is shown in the *lower panel* with values representing the mean of the three independent biological replicates ± standard deviations. *p* values were determined using a two-tailed unpaired Student's *t* test. ∗∗*p* < 0.01, ∗∗∗*p* < 0.001.

To further map the regions of *sod1* important for zinc-dependent changes in Sod1, we generated constructs to express the *sod1* ORF, including the *sod1* 3′UTR, from the *pgk1* promoter (pPgk1sod1sod1) (Fig. 7*A*). In *sod1*Δ expressing pPgk1sod1sod1, Sod1 protein abundance was significantly lower in cells grown in low-zinc conditions (Fig. 7*B*). Together, these results demonstrate that the *sod1* ORF and the *sod1* 3′UTR are sufficient for zinc-dependent changes in Sod1 protein abundance. Since the regulation was dependent on the ORF, we created a construct in which the *sod1* ORF from *S. pombe* was replaced with the *sod1* ORF from *S. cerevisiae*. When introduced into the *sod1* deletion strain, this construct revealed that *S. cerevisiae* Sod1 protein levels were also regulated by zinc (Fig. S2). These findings suggest that features of the *sod1* ORF critical for zinc-dependent regulation are conserved across yeast species.

### Ccr4 and Caf1 are required for growth under low-zinc conditions

**Figure 8:**
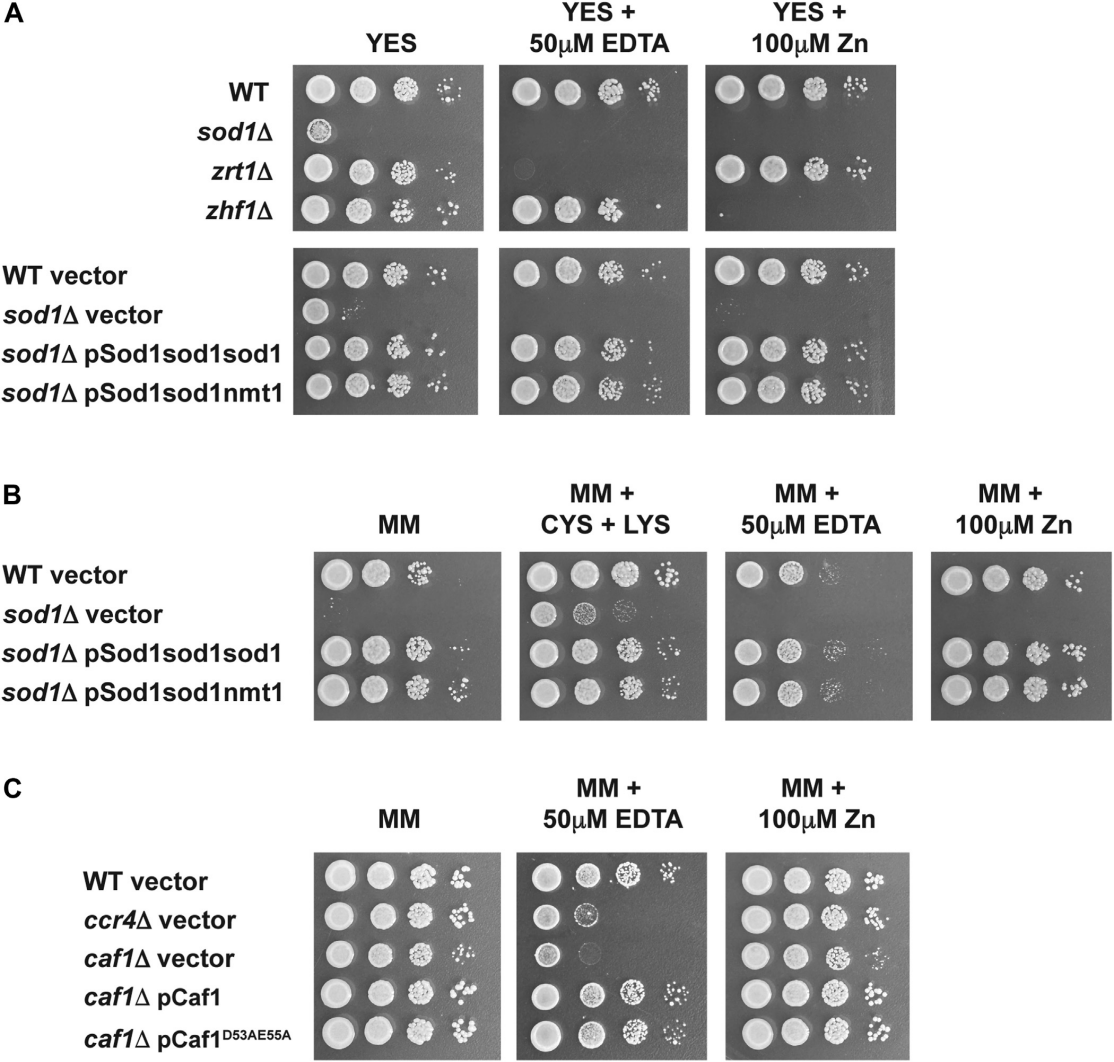
**Ccr4 and Caf1 are required for growth under low zinc conditions.***A*, the indicated strains were pre-grown overnight in YES medium. Cells were diluted to an OD^600^ of 1.0 and 10-fold serial dilutions plated on YES medium with the indicated supplements. *B*, the indicated strains were pre-grown overnight in EMM containing the appropriate auxotrophic requirements and lysine and cysteine. Cells were then washed in EMM without amino acids supplements, before being diluted to an OD^600^ of 1.0 and 10-fold serial dilutions plated onto PGM with the indicated supplements. *C*, the indicated strains were pre-grown overnight in PGM and were plated as described in Panel B. All Plates were incubated for 3 to 5 days at 31 °C before photography.

Given that zinc deficiency causes increased oxidative stress (37), and Sod1 has a well-established role as an antioxidant (38, 39), why would cells lower the levels of Sod1 under low zinc conditions? Studies in *S. cerevisiae* revealed that zinc deficiency leads to global changes in gene expression which reduce the levels of abundant non-essential zinc proteins (40). The net outcome of these changes is that the total number of protein zinc binding sites in the cell is nearly halved, thereby sparing zinc for essential proteins and functions (1). Since Cu-Zn superoxide is one of the most abundant zinc proteins in yeast (1), we hypothesized that the reduction in *sod1* mRNA and protein levels under low-zinc conditions was part of a zinc-sparing response. To test this hypothesis, we compared growth of *sod1*Δ cells expressing either the zinc regulated pSod1-sod1-sod1 plasmid or the constitutively expressed pSod1-sod1-nmt1 plasmid under low and high zinc conditions. Our rationale for this experiment was that increased expression of Sod1 under low-zinc conditions would deplete the total pool of zinc ions available for essential proteins, and therefore be inhibitory to growth. To test our hypothesis, we plated *sod1*Δ cells on YES medium supplemented with 50 μM EDTA or 100 μM zinc. As controls we also plated *zrt1*Δ cells, which are unable to grow under low-zinc conditions, and *zhf1*Δ cells, which are unable to grow under high-zinc conditions (41). *sod1*Δ cells had a strong growth defect on YES medium and were unable to grow in the presence of 50 μM EDTA or 100 μM zinc (Fig. 8*A*). All growth defects were rescued to the same extent by pSod1-sod1-sod1 and pSod1-sod1-nmt1. We observed similar results in minimal medium (MM), a more restrictive growth medium where *sod1*Δ cells are only able to grow in the presence of a lysine and cysteine supplement (42, 43) (Fig. 8*B*). Taken together, these results indicate that increased expression of *sod1* under low-zinc conditions is not inhibitory to growth.

Since deletion of *caf1* or *ccr4* leads to increased expression of *sod1* and potentially other mRNAs and proteins under low-zinc conditions, we tested whether these genes were required for growth in the presence of EDTA (Fig. 8*C*). Deletion of *caf1* led to a growth defect on EMM plates with an EDTA supplement, which was rescued by the introduction of pCaf1 and pCaf1^D53AE55A^. Deletion of *ccr4* also led to a growth defect under low-zinc conditions. This growth defect was milder than in *caf1*Δ, consistent with the deletion of *caf1* resulting in the additional loss of Ccr4 from the CCR4-NOT complex. These results demonstrate that Caf1 and Ccr4 are required for growth under low-zinc conditions.

## Discussion

Previous studies in fission yeast have shown that Sod1 is required for survival when cells are exposed to toxic (1.5 mM) concentrations of zinc (44). Here we show that *sod1* mRNA and protein levels are reduced in response to zinc deficiency and that this regulation occurs in a manner that is dependent on the Caf1 and Ccr4 deadenylases of the CCR4-NOT complex.

How could zinc status affect *sod1* gene expression in a manner that depends on Caf1 and Ccr4? The CCR4-NOT complex can regulate gene expression at multiple cellular levels including regulating chromatin remodeling, transcription initiation and elongation, mRNA quality control and nuclear export, and translation (30). However, its best characterized role is in facilitating deadenylation-dependent mRNA decay (45, 46). Given that the regulation of *sod1* is dependent on the Caf1 and Ccr4 deadenylases of the CCR4-NOT complex, one potential mechanism for its regulation by zinc is targeted deadenylation where the CCR4-NOT complex is specifically recruited to the *sod1* 3′UTR by an RNA-binding protein under low-zinc conditions. Multiple RNA-binding proteins are known which specifically target mRNAs in response to trace metal deficiency. These include the well-characterized mammalian IRP1 and IRP2, as well as Cth1 and Cth2 from *S. cerevisiae*, all of which target mRNAs under low-iron conditions (47, 48, 49). While there are examples in plants and animals of mRNAs whose stability is altered in response to cellular zinc status (16, 50, 51), to the best of our knowledge, there are no known RNA-binding proteins that specifically target mRNAs under low-zinc conditions. Further studies mapping the *sod1* 3′UTR could help determine if a specific RNA motif or structure is important, and if a zinc-responsive RNA-binding protein is required for regulation.

We found that the *sod1* ORF and 3′UTR are both necessary for the zinc-dependent changes in Sod1 protein abundance, suggesting that features of the *sod1* ORF and 3′UTR are critical for the regulation of Sod1 protein and potentially the *sod1* mRNA. In fission yeast, *adh1* gene expression is reduced under low zinc conditions in a manner that depends on the genomic region downstream of *adh1* (23). At this gene locus, gene repression results directly from a Loz1-regulated antisense transcript that is initiated downstream of the *adh1* gene under zinc-limiting conditions and runs through the entire gene and promoter region (19, 23). While the regulation of Sod1 differs from *adh1* in that it is independent of Loz1, natural antisense transcripts can act at multiple levels to alter gene expression, including altering the stability and translation of the sense mRNA (52). While we cannot rule out an antisense-based mechanism that requires Ccr4 and Caf1, we have not detected *sod1* antisense transcripts in low or high zinc conditions (23). Additionally, none have been identified in global transcriptomic analyses in fission yeast RNA processing mutants or under other stress conditions (53).

A potential way in which the ORF sequence could impact translation and deadenylation rates is through codon optimality (54). Factors that affect codon optimality include tRNA abundance, the levels of tRNAs relative to near cognate tRNAs, and wobble base pairing (32, 55). So far, the effects of zinc deficiency on codon optimality have yet to be examined. However, multiple proteins that are involved in tRNA modifications (*e.g.* Trm13), tRNA 3′ end processing (*e.g.* Rpc11), and wobble base pairing (*e.g.* Tad2) require a zinc cofactor (25). Some aminoacyl-tRNA synthetases also bind zinc, and targeted mutations disrupting zinc binding, as well as zinc chelation, both lead to reduced activity (56, 57, 58). An alternative explanation for the zinc-dependent changes in *sod1* mRNA levels is that zinc deficiency affects the activity of one or more of these enzymes, which in turn alters the composition of the tRNA pool. Based on codon usage within an mRNA, this could lead to slower rates of translation and enhanced CCR4-NOT dependent decay of specific transcripts under low-zinc conditions.

If regulation of *sod1* mRNA levels in response to zinc is a result of codon optimality, the respective contributions of Caf1 and Ccr4 may differ. Studies using a fully reconstituted CCR4-NOT complex from *S. pombe* revealed that Caf1 selectively deadenylates transcripts with low codon optimality and lower rates of translation elongation, whereas Ccr4 deadenylates all transcripts (54). Based on these observations, if codon optimality is important, a straightforward prediction would be that Caf1 and Ccr4 would facilitate the degradation of the *sod1* mRNA under low zinc conditions, whereas degradation in high zinc conditions would be dependent upon Ccr4. Another factor that could influence the net contributions of Caf1 and Ccr4 in the regulation is whether Caf1 activity is regulated by zinc *in vivo*. Caf1 is a DEDDh-type nuclease that requires Mg and potentially Mn for its activity (59). *In vitro* analyses examining cofactor requirements of Caf1 from *S. pombe* found that zinc ions outcompete Mg ions and inhibit Caf1 activity (59), whereas studies of a constitutive complex containing human CCR4 and CAF1 found that sub-micromolar concentrations of zinc stimulate Caf1 activity (60). At the mRNA level we found that the levels of *sod1* transcripts were comparable in wild type, *caf1*Δ pCaf1, and *caf1*Δ pCaf1^D53AE55A^ cells. While these results do not rule out a regulatory function for Caf1, they demonstrate that Ccr4 activity is sufficient for the regulation of *sod1* mRNA levels by zinc, indicating that the regulation is not specific to Caf1. We also found the *sod1* mRNA profiles in response to zinc of *ccr4*Δ differs from *caf1*Δ, and that *sod1* mRNA levels increased by ∼3-fold in the *ccr4* deletion strain without a corresponding increase at the protein level. We do not yet know why *sod1* mRNAs accumulate to higher levels with the deletion of *ccr4* compared to *caf1*. However, given that any Caf1-dependent effect could be masked by the presence of Ccr4, these results raise the possibility that Caf1 may directly regulate Sod1 protein abundance. To clarify these results, future experiments should focus on testing additional aspects of zinc-dependent *sod1* expression, such as measuring zinc-dependent mRNA decay rates and translation rates in cells with inactive Caf1 and Ccr4 alleles.

In yeast, zinc deficiency reduces the expression of other abundant zinc-binding proteins, such as alcohol dehydrogenase 1, as part of a zinc-sparing response (49, 59). To determine whether the regulation of *sod1* expression by zinc is also part of this response, we tested whether increased *sod1* expression under low-zinc conditions would cause a growth defect. We found that increased *sod1* expression did not inhibit growth, possibly because the increase was insufficient to significantly affect the intracellular labile zinc pool. Alternatively, higher *sod1* expression in low zinc may have resulted in the accumulation of unmetallated Sod1 within cells (1). While this could potentially lead to elevated levels of unfolded Sod1 protein, yeast upregulate protein chaperones during zinc deficiency to mitigate the increased burden of unfolded proteins (20, 61). We also found that Ccr4 and Caf1 are required for optimal growth under low-zinc conditions. Given the global role of these proteins in regulating gene expression, it is tempting to speculate that other zinc-binding proteins and their mRNAs may be specifically targeted by these deadenylases in low zinc conditions, and the resulting mis-regulation could contribute to the growth defect. In addition to zinc sparing, in *S. cerevisiae*, a zinc-binding, inactive allele of Sod1 was able to rescue a zinc-toxicity slow-growth phenotype of a *sod1* deletion strain (62). The regulation of Sod1 by zinc may therefore also serve to increase levels of zinc-binding proteins in high-zinc conditions, which act as a zinc sink for protection against zinc toxicity. The regulation of Sod1 protein levels by zinc may play a protective role for cells under both zinc deficiency and excess conditions. In *S. pombe*, as well as in other organisms such as *S. cerevisiae*, *Arabidopsis*, and *Sorghum*, Sod1 protein levels are reduced in response to zinc deficiency (24, 63, 64). This suggests that the zinc-dependent regulation of Sod1 may be an important aspect of zinc homeostasis in both the plant and fungal kingdoms.

## Experimental procedures

### Strain generation and growth conditions

The *S. pombe* strains used are listed in Table S1. Yeast strains *caf1*Δ and *sod1*Δ were generated by PCR-based gene replacements with the KanMX6 cassette (65). For these strains, the KanMX6 cassette was amplified using primers with 60 to 80 bp of homology to the target gene of interest. The resulting PCR products were then introduced into wild-type strain JW81 (h-ade6-M210 leu1-32 ura4-D18) using a lithium acetate transformation protocol with selection on YES with 100 μg/ml G418. Correct transformants were identified using diagnostic PCR. The strain *ccr4*Δ was also generated using the above approach with the exception that a ccr4-KO cassette was generated and amplified for the gene replacement. The ccr4-KO cassette contained the KanMX6 cassette flanked by 750 bp of DNA with sequence homology to the regions immediately upstream and downstream of the *ccr4* ORF.

Strains were grown at 31 °C with shaking in either yeast extract + supplements (YES; 0.5% Yeast Extract, 3% glucose and 225 mg/l uracil, leucine, and adenine), or a derivative of Edinburgh minimal medium (EMM) which lacks zinc (ZL-EMM). In experiments using ZL-EMM, strains were initially pre-grown to exponential phase in YES. Cells were then washed twice in ZL-EMM before inoculation to an OD^600^ of ∼0.3 in metal free tubes containing fresh ZL-EMM with or without ZnCl_2_. Cells were grown for a further 14 to 16 hrs before harvesting. Strains used for the growth assays performed in Fig. 8, *B* and *C*, were grown overnight in Pombe Glutamate medium (PGM) with the appropriate auxotrophic supplements. For the plate assays, cells were diluted to an OD^600^ of 1.0. Three 10-fold serial dilutions were then performed before cells were plated on PGM plates with the indicated, amino acid, EDTA and Zn^2+^ supplements.

### Plasmid construction

To generate pSod1^519^ and pSod1, the *sod1* gene with either 519 bp of the promoter (pSod1^519^) or 1786 bp of the promoter (pSod1) was amplified by PCR using primers containing KpnI/SacI sites for cloning. Following digestion with KpnI/SacI, PCR products were cloned into similar sites in pbluescript SK+ before being subcloned into similar sites in the vector JK148 (66). The additional plasmids used for mapping regions of the *sod1* gene required for zinc-dependent regulation are all derivatives of JK148. The plasmids were generated by PCR amplifying the respective promoter (*sod1* or *pgk1*), ORF (*sod1* or *GFP*), and terminator (*sod1* or Sc.*adh1*) regions with primers containing restriction sites to facilitate cloning. Digested PCR fragments were then sequentially cloned into the vector JK148. In the plasmids, promoters were introduced as KpnI/EcoRI fragments, ORFs as EcoRI/BamHI fragments, and terminator as BamHI/SacI or BamHI/AscI fragments. The plasmid pCaf1 was generated by amplifying the entire *caf1* gene (including its promoter and terminator) using primers that contained BamHI and EagI sites. The resulting PCR product was digested with BamHI and EagI and cloned into similar sites in the vector JK148. To generate a plasmid expressing the catalytically inactive pCaf1^D53AE55A^ allele, the *caf1* gene from pCaf1 was first subcloned as an EagI/BamHI fragment into pbluescript SK+. Two sequential rounds of Quikchange site-directed mutagenesis were then performed to introduce the D53A and E55A substitutions. After sequence confirmation to confirm the introduction of the substitutions, the Caf1^D53AE55A^ fragment was subcloned as an EagI/BamHI into JK148. Clones were confirmed using DNA sequencing analysis. All JK148 plasmids were linearized with NruI to target their integration to *leu1*.

### RNA preparation and RNA blots

Total RNA was harvested from 5-10ml of cells using the acidic phenol method. RNA concentration was determined using NanoDrop OneC (Thermo Fisher Scientific). RNA blots were run using a 1% agarose gel containing 3.7% formaldehyde buffered with 1X MOPS buffer (20 mM MOPS pH 7.0, 2 mM sodium acetate, 1 mM EDTA). RNA was loaded at a concentration of ∼5 to 15 μg depending on the abundance of the transcript of interest, with ∼4 μl RNA loading dye (76% formamide, 1X MOPS buffer, 1X loading dye, 2% EtBr). Gels were run for 4 hours at 75V, washed twice in ddH2O before transferring to a nylon membrane (Invitrogen BrightStarTM-Plus) by capillary transfer in an alkaline transfer buffer (3M NaCl/0.01 M NaOH). Following transfer, membranes were washed for 2 to 3 min in 6x SSC (0.9M NaCl, 0.09 M sodium citrate dihydrate) and RNA crosslinked to the membrane using a UV crosslinker. Membranes were probed with radiolabeled, strand-specific RNA probes created using a MAXIscript T7 kit (Invitrogen). Probes were hybridized to membranes overnight at 60 °C in Northern hybridization buffer (0.5 M sodium phosphate pH 7.2, 7% SDS, 1 mM EDTA). Blots were washed in 2X SSC/0.1% SDS and exposed to a phosphor screen 21 (Amersham Biosciences/GE Healthcare Life Sciences). RNAs were detected using a GE Typhoon FLA 9500 using ImageJ software.

### Protein preparation and immunoblot analysis

For immunoblot analyses protein lysates were prepared using the trichloroacetic acid (TCA) method as described previously (21) with the exception that tubes were vortexed for 2 to 10 rounds of 30 seconds each with at least 2 minutes on ice in between each vortex. Protein extracts were separated by SDS-PAGE analysis before transferring to a nitrocellulose membrane by immunoblotting. Immunoblots were probed with anti-Sod1 (Sigma-Aldrich HPA 001401), anti-GFP (Sigma-Aldrich C3956), and anti-Act1 (abCAM ab170325) primary antibodies followed by IRDye800CW-conjugated anti-mouse IgG (LI-COR) and IRDye680-conjugated anti-rabbit IgG (LI-COR) secondary antibodies. Signal intensities were analyzed using the Odyssey IR imaging System (LICOR).

## Data availability

All data is included in the article.

## Supporting information

This article contains supporting information. The following references are cited in the supporting information (19, 23, 67, 68).

## Conflicts of interest

The authors declare that they have no conflicts of interest with the contents of this article.
