# Supplementary material for: Regulation of sod1 mRNA and protein abundance by zinc in fission yeast is dependent on the CCR4-NOT complex
Source: J Biol Chem. 2025 Jan 4;301(2):108156. doi: 10.1016/j.jbc.2025.108156 (PMC11830320; doi:10.1016/j.jbc.2025.108156)
Supplement: Supporting Materials_Figures [file mmc1.docx]

**Supplemental Figures**

**­­**

**Figure S1. *sod1-lacZ* reporter activity is not regulated by zinc.**

Wild-type cells with the empty vector or *sod1-lacZ*, *zrt1-lacZ,* or *adh1-lacZ* reporters were grown overnight in ZL-EMM with 0 or 100 μM Zn^2+^ and β-galactosidase activity measured. The *zrt1-lacZ* reporter is repressed by Loz1 in high zinc conditions, and the *adh1-lacZ* reporter is not regulated by zinc status. The results show the mean of three independent biological replicas ± standard deviations. *p* values were determined using two-tailed unpaired Student's *t* test. *** p < 0.001


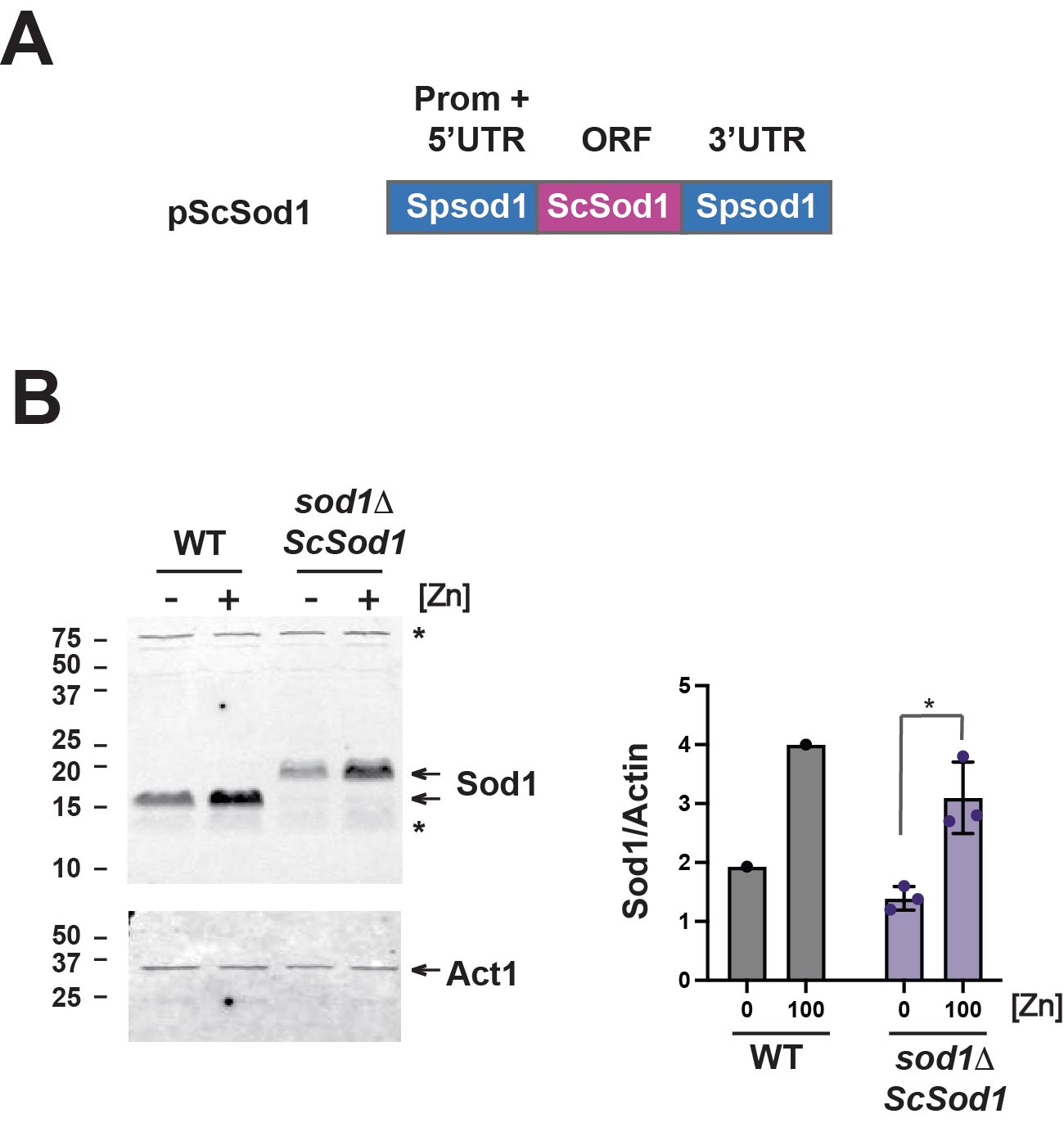


**Figure S2. Expression of Sod1 from *S. cerevisiae* in *S. pombe*.**

(A) Schematic diagram of the pScSod1 plasmid. The plasmid contains the *sod1* ORF from *S. cerevisiae* with the fission yeast promoter, 5’UTR and 3’UTR. (B) The pScSod1 plasmid was integrated into the genome of *S. pombe* *sod1*Δ to generate *sod1*Δ ScSod1. Immunoblots were performed with total protein extracts from wild-type cells with the empty vector and *sod1*Δ ScSod1 grown in zinc-limiting EMM (ZL-EMM) supplemented with 0 or 100 µM zinc. Immunoblots were hybridized with antibodies to Sod1 (arrow) and loading control Actin (Act1). Non-specific bands are marked with an asterisk. Molecular weight markers in KDa are indicated on the left. The quantification of 3 independent blots with Sod1 signal normalized to Actin is shown. The values representing the mean of the three independent ScSod1 biological replicas ± standard deviations. *p* values were determined using two-tailed unpaired Student's *t* test. * *p* < 0.05. Currently, it is unclear why the ScSod1 protein is approximately 20 kDa, larger than the expected 16 kDa. However, *sod1*Δ ScSod1 grew in ZL-EMM, which lacks lysine and methionine supplements, indicating that it is functional.
