# Supplementary material for: Regulation of sod1 mRNA and protein abundance by zinc in fission yeast is dependent on the CCR4-NOT complex
Source: J Biol Chem. 2025 Jan 4;301(2):108156. doi: 10.1016/j.jbc.2025.108156 (PMC11830320; doi:10.1016/j.jbc.2025.108156)
Supplement: Supporting Materials_Table_1 [file mmc2.docx]

**Supporting Information**

**Table S1. *S. po­mbe* strains used in this study.**

| **Strain** | **Genotype** | **Reference** |
| --- | --- | --- |
| JW81 | *h- ade6-M210 leu1-32 ura4-D18* | (1) |
| *loz1*Δ | *h+ ade6-M210 leu1-32 ura4-D18 SPAC25B8.19c*Δ::*kan^R^* | Bioneer |
| *atf1*Δ | *h- ade6-M210 atf1-D15*::*ura4 ura4-D18 his3-D1 leu1-32* | (2) |
| *pap1*Δ | *h+ ade6-M210 leu1-32 ura4-D18 SPAC1783.07c::kan^R^* | Bioneer |
| *sty1*Δ | *h+ ade6-M210 leu1-32 ura4-D18 SPAC24B11.06c::kan^R^* | Bioneer |
| JW81 JK148 | *h- ade6-M210 leu1-32 ura4-D18 JK148::leu1^+^* | (3) |
| *zrt1*Δ | *h- ade6-M210 leu1-32 ura4-D18 SPBC16D10.06*::*kan^R^* | (4) |
| *zhf1*Δ | *h+ ade6-M210 leu1-32 ura4-D18 SPAC23C11.14*::*kan^R^* | Bioneer |
|  | | |
| *sod1*Δ | *h- ade6-M210 leu1-32 ura4-D18 SPAC821.10c*Δ::*kan^R^* | This study |
| *sod1*Δ pSod1^519^ | *h- ade6-M210 leu1-32 ura4-D18 SPAC821.10c*Δ::*kan^R^* psod1^519^*::leu1^+^* | This study |
| *sod1*Δ pSod1 | *h- ade6-M210 leu1-32 ura4-D18 SPAC821.10c*Δ::*kan^R^* psod1*::leu1^+^* | This study |
| *caf1*Δ | *h- ade6-M210 leu1-32 ura4-D18 SPCC18.06c*Δ::*kan^R^* | This study |
| *caf1*Δ JK148 | *h- ade6-M210 leu1-32 ura4-D18 SPCC18.06c*Δ::*kan^R^ JK148::leu1^+^* | This study |
| *caf1*Δ pCaf1 | *h- ade6-M210 leu1-32 ura4-D18 SPCC18.06c*Δ::*kan^R^ pcaf1::leu1^+^* | This study |
| *caf1*Δ pCaf1^D53AE55A^ | *h- ade6-M210 leu1-32 ura4-D18 SPCC18.06c*Δ::*kan^R^ pcaf1* ^D53AE55A^*::leu1^+^* | This study |
| *ccr4*Δ | *h- ade6-M210 leu1-32 ura4-D18 SPCC31H12.08c*::*kan^R^* | This study |
| *ccr4*Δ JK148 | *h- ade6-M210 leu1-32 ura4-D18 SPCC31H12.08c*::*kan^R^ JK148::leu1^+^* | This study |
| *sod1*Δ pSod1sod1sod1 | *h- ade6-M210 leu1-32 ura4-D18 SPAC821.10c*Δ::*kan^R^ psod1sod1sod1::leu1^+^* | This study |
| *sod1*Δ pSod1sod1nmt1 | *h- ade6-M210 leu1-32 ura4-D18 SPAC821.10c*Δ::*kan^R^ psod1sod1nmt1::leu1^+^* | This study |
| *sod1*Δ pPgk1GFPsod1 | *h- ade6-M210 leu1-32 ura4-D18 SPAC821.10c*Δ::*kan^R^ ppgk1GFPsod1::leu1^+^* | This study |
| *sod1*Δ pPgk1GFPadh1 | *h- ade6-M210 leu1-32 ura4-D18 SPAC821.10c*Δ::*kan^R^ ppgk1GFPadh1::leu1^+^* | This study |
| *sod1*Δ pSod1GFPsod1 | *h- ade6-M210 leu1-32 ura4-D18 SPAC821.10c*Δ::*kan^R^ p* *sod1GFPsod1::leu1^+^* | This study |
| *sod1*Δ pPgk1sod1sod1 | *h- ade6-M210 leu1-32 ura4-D18 SPAC821.10c*Δ::*kan^R^ p* *pgk1sod1sod1::leu1^+^* | This study |

**References**

1. Wu, J. Q., Kuhn, J. R., Kovar, D. R., and Pollard, T. D. (2003) Spatial and temporal pathway for assembly and constriction of the contractile ring in fission yeast cytokinesis. *Dev Cell* **5**, 723-734

2. Kon, N., Krawchuk, M. D., Warren, B. G., Smith, G. R., and Wahls, W. P. (1997) Transcription factor Mts1/Mts2 (Atf1/Pcr1, Gad7/Pcr1) activates the M26 meiotic recombination hotspot in Schizosaccharomyces pombe. *Proc Natl Acad Sci U S A* **94**, 13765-13770

3. Ehrensberger, K. M., Mason, C., Corkins, M. E., Anderson, C., Dutrow, N., Cairns, B. R., Dalley, B., Milash, B., and Bird, A. J. (2013) Zinc-dependent regulation of the Adh1 antisense transcript in fission yeast. *J Biol Chem* **288**, 759-769

4. Corkins, M. E., May, M., Ehrensberger, K. M., Hu, Y. M., Liu, Y. H., Bloor, S. D., Jenkins, B., Runge, K. W., and Bird, A. J. (2013) Zinc finger protein Loz1 is required for zinc-responsive regulation of gene expression in fission yeast. *Proc Natl Acad Sci U S A* **110**, 15371-15376
